# Development and Grasp Stability Estimation of Sensorized Soft Robotic Hand

**DOI:** 10.3389/frobt.2021.619390

**Published:** 2021-03-31

**Authors:** P. M. Khin, Jin H. Low, Marcelo H. Ang, Chen H. Yeow

**Affiliations:** ^1^Advanced Robotics Centre, National University of Singapore, Singapore, Singapore; ^2^Department of Mechanical Engineering, National University of Singapore, Singapore, Singapore; ^3^Department of Biomedical Engineering, National University of Singapore, Singapore, Singapore

**Keywords:** machine learning, one shot learning, pneumatic actuators, soft end effector, grasping

## Abstract

This paper introduces the development of an anthropomorphic soft robotic hand integrated with multiple flexible force sensors in the fingers. By leveraging on the integrated force sensing mechanism, grip state estimation networks have been developed. The robotic hand was tasked to hold the given object on the table for 1.5 s and lift it up within 1 s. The object manipulation experiment of grasping and lifting the given objects were conducted with various pneumatic pressure (50, 80, and 120 kPa). Learning networks were developed to estimate occurrence of object instability and slippage due to acceleration of the robot or insufficient grasp strength. Hence the grip state estimation network can potentially feedback object stability status to the pneumatic control system. This would allow the pneumatic system to use suitable pneumatic pressure to efficiently handle different objects, i.e., lower pneumatic pressure (50 kPa) for lightweight objects which do not require high grasping strength. The learning process of the soft hand is made challenging by curating a diverse selection of daily objects, some of which displays dynamic change in shape upon grasping. To address the cost of collecting extensive training datasets, we adopted one-shot learning (OSL) technique with a long short-term memory (LSTM) recurrent neural network. OSL aims to allow the networks to learn based on limited training data. It also promotes the scalability of the network to accommodate more grasping objects in the future. Three types of LSTM-based networks have been developed and their performance has been evaluated in this study. Among the three LSTM networks, triplet network achieved overall stability estimation accuracy at 89.96%, followed by LSTM network with 88.00% and Siamese LSTM network with 85.16%.

## Introduction

The human body consists of many complex and fascinating organs like the hand. The multi-hinged, multi-fingered hand enables one to carry out complex manipulation tasks. It is also integrated with numerous sensory modules to enable somatosensory perception and proprioception. The perceptual information shapes the cognitive ability, which allows a person to feel and learn about objects in his environment. Inspired by the structure and sensing capabilities of the hand, this paper presents the development of a sensorized, anthropomorphic soft robotic hand which is composed of soft fabric-based pneumatic actuators and flexible force sensor arrays.

It has been demonstrated that embedded tactile sensing capabilities allow a soft robotic hand to feel the physical features such as shapes, size and stiffness, of the objects that it is interacting with (Zhao et al., 2016; Chen et al., 2018). This paper attempts to utilize the embedded force sensing mechanism to learn to recognize the stability of objects which have been grasped by the sensorized soft hand. As the field of soft robotics merges with machine learning, data-driven capabilities of soft robots, not exclusive to soft hands, have been developed i.e., texture recognition (Sankar et al., 2020), proprioception feedback (Thuruthel et al., 2019) and slippage detection (Shirafuji and Hosoda, 2014). On its own, it has been demonstrated that soft robotic hands are able to grasp a large variety of objects (Deimel and Brock, 2015; She et al., 2016). However, scalability of learnt networks is an issue which has yet to be addressed. It is noted that collecting training data for a large range of objects that the soft hand can potentially grasp, is tedious and time consuming. In this respect, one shot learning (OSL) technique is adopted in the development of networks which learn to estimate the stability of grasped object. This enhances the potential of scaling up the network to accommodate larger variety of objects in the future.

This paper introduces the development of fabric based anthropomorphic soft robotic hand. By leveraging on the integrated force sensors, this paper aims to contribute by developing one shot learning based grip state estimation networks which interpret the stability of objects that have been grasped by the soft hand. It is arranged in the following order. Section Related Works covers related works. Section Development of Sensorized Soft Robotic Hand describes the methods to fabricate the actuators and sensors. It also details the characterization experiments of the soft robotic hand. Section Grip State Estimation Networks and Experiments describes the data collection experiments and development of grip state estimation networks. Section Performance Evaluation includes the results and discussion of the experiments. Section Conclusion concludes the paper.

## Related Works

To date, numerous robotic hands have been developed in both commercial and academic research, traditionally using a combination of alloys and metals. In the alternate paradigm of robotic hands, mechanically softer robots have been developed using elastomeric materials. Intrinsically compliant nature of these robots seeks to emulate the properties of biological tissues (Godfrey et al., 2013; Tavakoli and Almeida, 2014; Deimel and Brock, 2015). Actuators of this genre can be fabricated using different methods such as shape deposition manufacturing (Deimel and Brock, 2015) and 3D-printing (Yap et al., 2016). These soft actuators can be activated using compressed fluid (Galloway et al., 2016), heat (She et al., 2015), or cables (Renda et al., 2014). In comparison to their rigid counterparts, soft robotic hands have a higher tendency to conform to the shapes of external objects. Moreover, the soft actuators are also capable of high payload to weight ratio (Chen et al., 2018).

Rich diversity of flexible sensing technologies that can be integrated in soft robots have been shared in the literature, i.e., piezoelectric polyvinylidenefluoride (PVDF) film-based sensor, force sensors, strain gauge sensor sensors and waveguide sensors (Shirafuji and Hosoda, 2014; Homberg et al., 2015, 2018; Zhao et al., 2016; Shih et al., 2017; Wall et al., 2017; Rocha et al., 2018, Truby et al., 2018, Choi et al., 2018). Integrated sensing is useful in unstructured environment (Kazemi et al., 2014) or in environment with low visibility (Liarokapis et al., 2015). Due to the structural material and operation mechanism, the hand provides compliant interface and sufficient strength to grasp a variety of daily objects. However, based on the physical features such as stiffness and weight, different objects require different grasping strength.

Threshold-based slip detection (Ponraj et al., 2019) was reported using sensorized tendon driven modular gripper. However, most of the slip detection works were developed and demonstrated with limited test objects, i.e., water bottle (Nakagawa-Silva et al., 2019) and two-dimensional rigid boards (Jamali and Sammut, 2012; Meier et al., 2016). This limits their applicability to different daily objects that can be handled by the soft robotic hand as daily objects come in a diverse range of shape, size, weight and stiffness. To cope with the large variability in force profiles, neural networks can be used to detect the slippage of various objects. An artificial neural network (ANN) was developed by Shirafuji and Hosoda to detect slippage from stress measured by the strain gauge integrated in soft fingers (Shirafuji and Hosoda, 2014).

This paper attempts to take the data-driven approach to learn to recognize stability of objects which have been grasped by the sensorized soft hand. During grasping and lifting motion of soft robotic hand, the object may experience tilting or slight shifting in position due to grasping strength of the soft hand or due to acceleration of robot manipulator. If a heavy object is grasped with insufficient strength, the object may tilt and proceed to slip from the hand as the robotic hand attempts to lift it up. However, large datasets would be required to train a neural network that effectively recognizes the grasp instability of different objects. Collecting extensive training dataset for each object can be time consuming and tedious. Moreover, the number of objects that can be grasped by the robotic hand may vary as the design of the hand evolves. Hence, there is a concern of training datasets exponentially increasing with the number of grasping objects. Therefore, this paper takes OSL based approach. OSL requires fewer training data (Fei-Fei et al., 2006) and hence, the prior concern of deep neural network requiring extensive datasets is addressed. OSL has been implemented in tactile-based recognition network for object classification (Kaboli et al., 2016). As compared to vision, OSL has not received much attention in tactile recognition, much less in sensorized soft robotics (Abderrahmane et al., 2020). Due to lightweight training dataset, this learning approach also allow us to potentially scale up the training process in the future to include more variety of objects.

## Development of Sensorized Soft Robotic Hand

### Fabrication of Anthropomorphic Soft Robotic Hand

**Figure 1:**
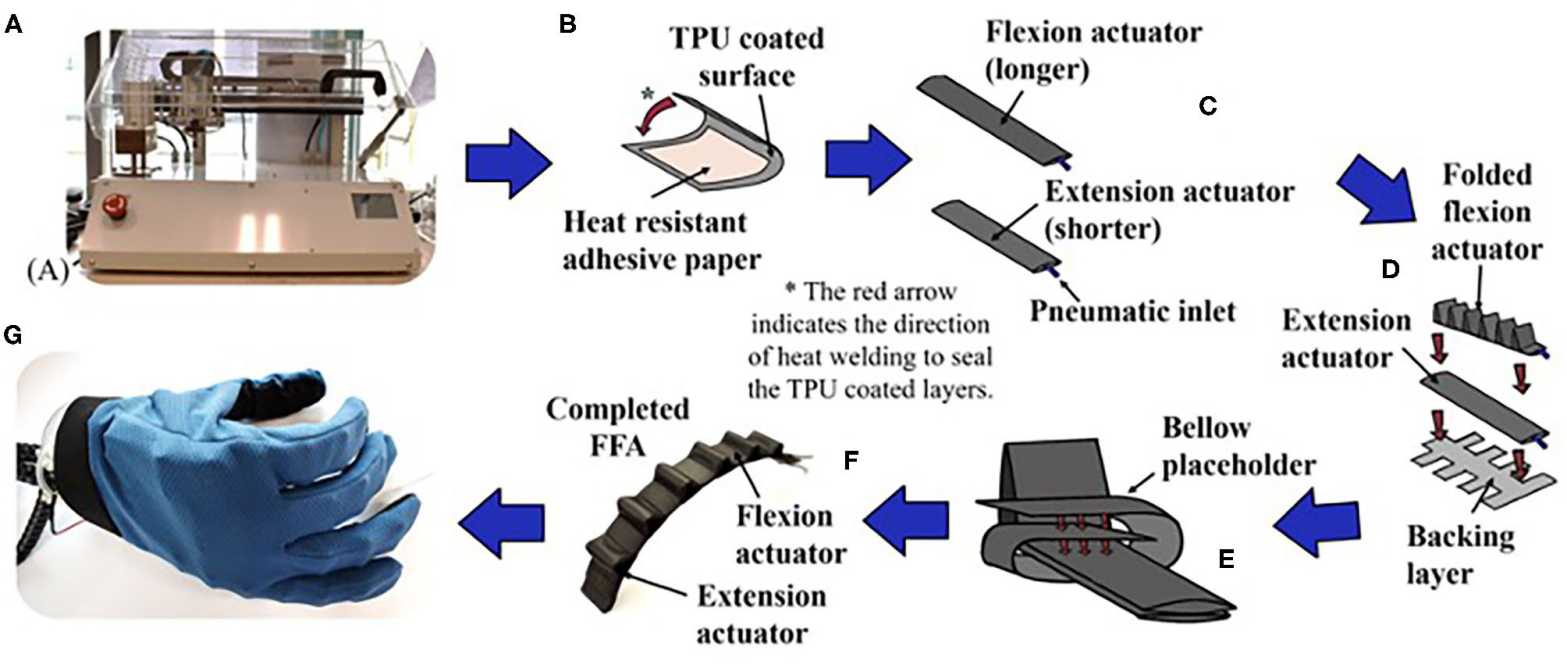
Illustration of the fabrication stages of FFA. **(A)** Custom heat jig machine, **(B–F)** fabrication cycle of one FFA, **(G)** assembled FFA in soft hand.

Fabric-based finger actuators (FFAs) of the robotic hand were made of TPU-coated nylon N420D fabric (Jiaxing Inch ECO Materials Co., LTD, Zhejiang, China). Airtight pneumatic structures could be achieved with heat-press-sealing based approach, which was reported in our previous work (Low et al., 2017). In this paper, we built a custom heat-jig machine to fully automate and standardize the fabrication protocol (Figure 1A). We also modified the sealing process to achieve pneumatic channels of maximum width without having to compromise on the overall width of the actuator.

To fabricate the pneumatic bladder of the FFA, the first step was to delineate the layout of the inner pneumatic channel by patterning with a layer of high temperature resistant adhesive paper on the TPU-coated surface of the fabric (Figure 1B). During the subsequent sealing step, this prevented TPU in the delineated region from sealing together and an inner pneumatic pocket was created in the adjacent layer of welded fabric pieces. A 5 mm width sealing area was reserved at the top and bottom of the air channel, while a 22 mm width sealing area was reserved at the side.

The second step would be to seal the pneumatic bladder. The fabric was double-folded into a sleeve and the edges were heat sealed by the heat-jig (Figure 1C). The open ends were sealed to form two closed ends, one of which was fitted with a tube. One polyurethane air tube of 52 mm diameter was used as an inlet of choice for the pneumatic bladder. This created a fully sealed bladder with a channel width of 22 mm, which could be used to create FFA. One short pneumatic bladder, one longer pneumatic bladder and fishbone shaped fabric backing (Figure 1D) were used to make the bladder into FFA.

FFA was created by folding the longer air bladder in a segmented sinuous pattern and securing it on top of the shorter bladder (Figure 1D). Upon actuation, the longer air bladder served as the flexion component, and the shorter air bladder served as the extension component. Instead of using cable-ties to secure the folds as mentioned previously (Low et al., 2017), the folded air bladder was secured in place using a backing layer of fishbone shaped TPU-coated fabric. The backing fabric allowed the folds to be secured at predefined regular intervals (Figure 1E). This enabled further reduction of the width of FFA, as additional sealing area was no longer required for the insertion of the cable-ties. Furthermore, we were able to construct FFA solely using fabric, except the polyurethane air tube, which was used as inlet of air source.

The length of the folds and the base of the folds were 38 and 25 mm, respectively. The actuation length of the FFA (Figure 1F) was defined as the sum of base of the folds. Three types of FFA with different folds and actuation lengths were proposed in this study (Table 1). The average weight of a FFA was ~16.8 g.

**Table 1:** Dimensions of FFAs used in this study.

**Finger**		**Folds**	**Actuations length (mm)**	**Actual finger length (mm)**
I	Pollex	4	100	105 ± 9.3
II	Digitus secundus manus	6	150	150 ± 16.6
III	Digitus medius manus	6	150	157 ± 14.7
IV	Digitus quartus manus	6	150	146 ± 15
V	Digitus minimus manus	150	125	124 ± 12.7

### Characterization of Anthropomorphic Soft Robotic Hand

Important characteristics of the FFA such as bending angle, compressive tip force and pull force were investigated. Each parameter corresponding to various air pressures, ranging from 0 to 120 kPa, was tested at incremental steps of 30 kPa. Three samples were used, and the average of the data was recorded at each pneumatic pressure.

**Figure 2:**
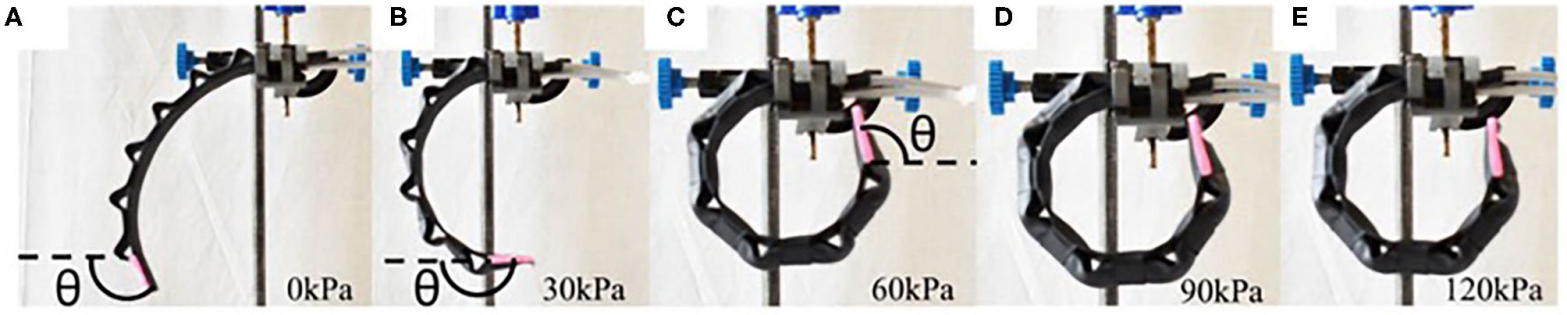
**(A–E)** Actuation profile of FFA at different pneumatic pressure.

Bending angle of the inflated actuator was analyzed with an image-processing program, ImageJ (National Institutes of Health, Bethesda, MD). The actuator was clamped on a retort stand with a red line on its uninflated tip (Figure 2). A DSLR camera was used to capture the image of the actuation and the image was post-processed with ImageJ to obtain the bending angle. The bending angle was measured by obtaining the angle between the horizontal x-axis and the line on the tip.

**Figure 3:**
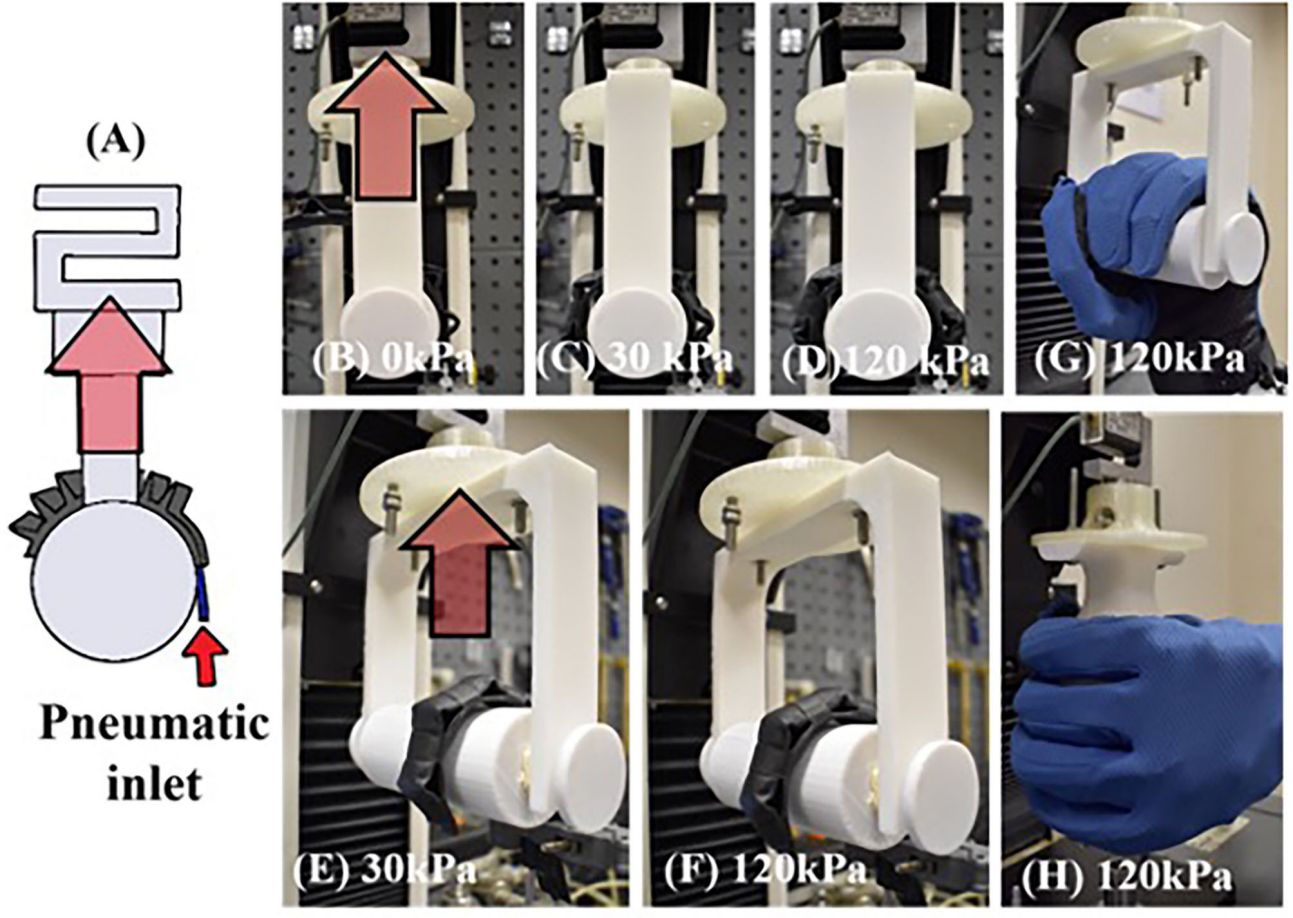
**(A)** Schematic diagram of grip test. **(B–F)** Photo of FFA in vertical grip test and soft robotic hand in **(G)** vertical, and **(H)** horizontal grip test.

To analyze the ability of FFA to grasp and hold, a pull test was conducted using the Instron Universal Tester 3345 (Instron, Norwood, MA) to measure the maximum vertical resistive grip force (Figure 3A). A test jig, consisting of a cylinder with diameter of 50 mm secured to the load cell of the Instron machine via a customized base, was designed for the pull test (Figures 3B–F). The maximum resistive grip force was defined as the maximum force needed to hold a cylinder in place as it was being pulled upward at a fixed velocity of 8 mm/s. The recorded force at the point of release was the maximum resistive grip force.

Similarly, the grip strength of the soft robotic hand (as described in Section Grip State Estimation Networks and Experiments) was evaluated by conducting the same pull test with two different grip conditions, (i) vertical grip (Figure 3G) and (ii) horizontal grip (Figure 3H). The test cylinder was positioned at the palm of the hand and the FFAs were pressurized to 120 kPa to fully enclose the cylinder. The cylinder was then pulled upward at a fixed velocity of 8 mm/s. The maximum vertical and horizontal resistive grip force were obtained according to the grip configurations, respectively. Two object sizes (50 and 75 mm) were tested in horizontal grip condition while one object size (50 mm) was tested in vertical grip.

### Fabrication of Force Sensor

**Figure 4:**
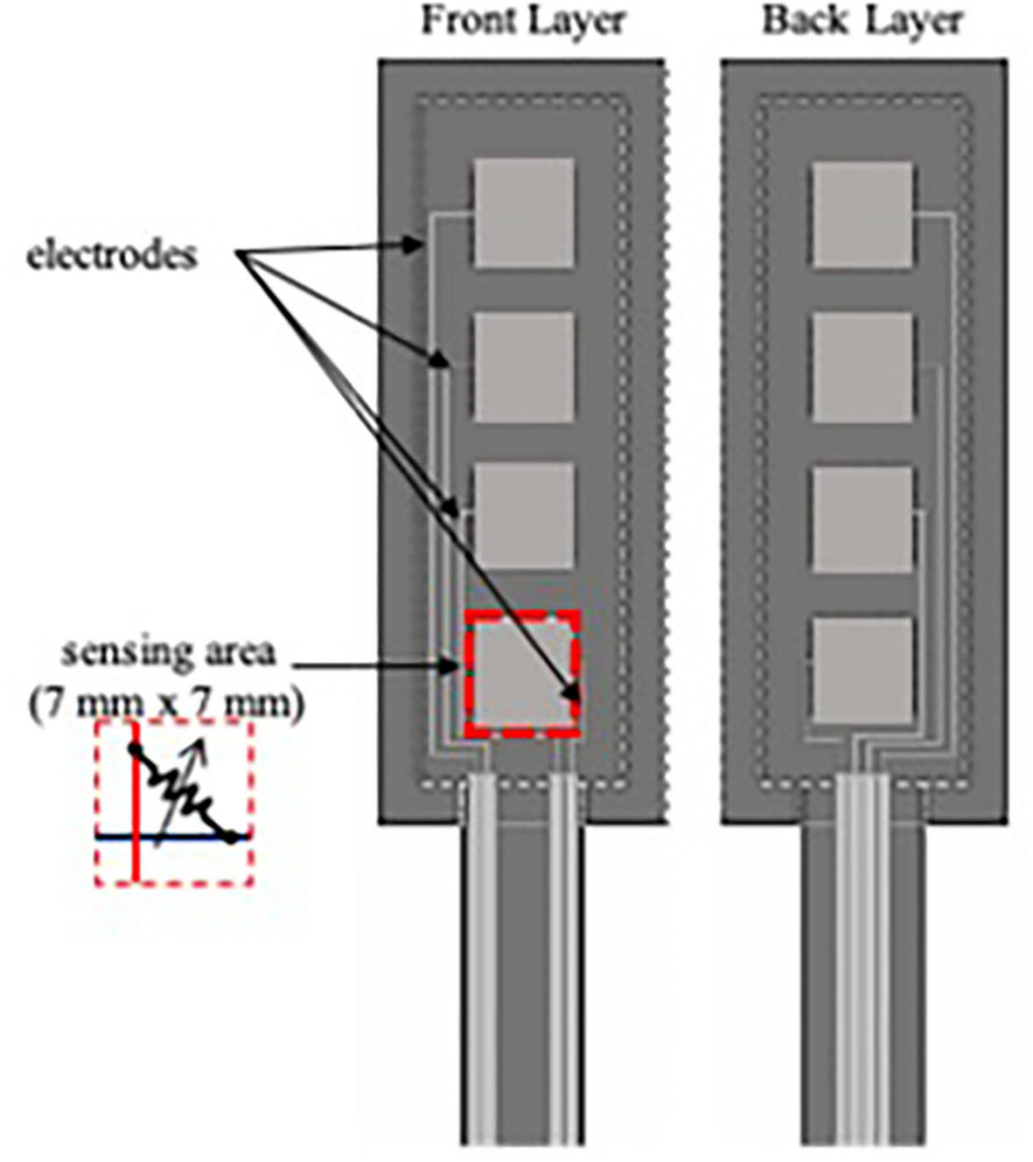
Schematic diagram of flexible force sensor array.

A force sensor array was made using layers of printed electrode on polyethylene sheets and piezoresistive fabric (Figure 4). Each array of piezoresistive sensor consisted of four taxels, each of which measured to be 7 mm by 7 mm. Spatial resolution between adjacent taxel was 4 mm. Due to changes in resistance upon application of force, voltage output of the sensor changed from its idle state. The extent of voltage drop depended on the applied load on the sensor. Five force sensor arrays, each of which has four sensing taxels, were fabricated and were embedded in each finger of the soft robotic hand.

## Grip State Estimation Networks and Experiments

### Data Collection Experiments

**Figure 5:**
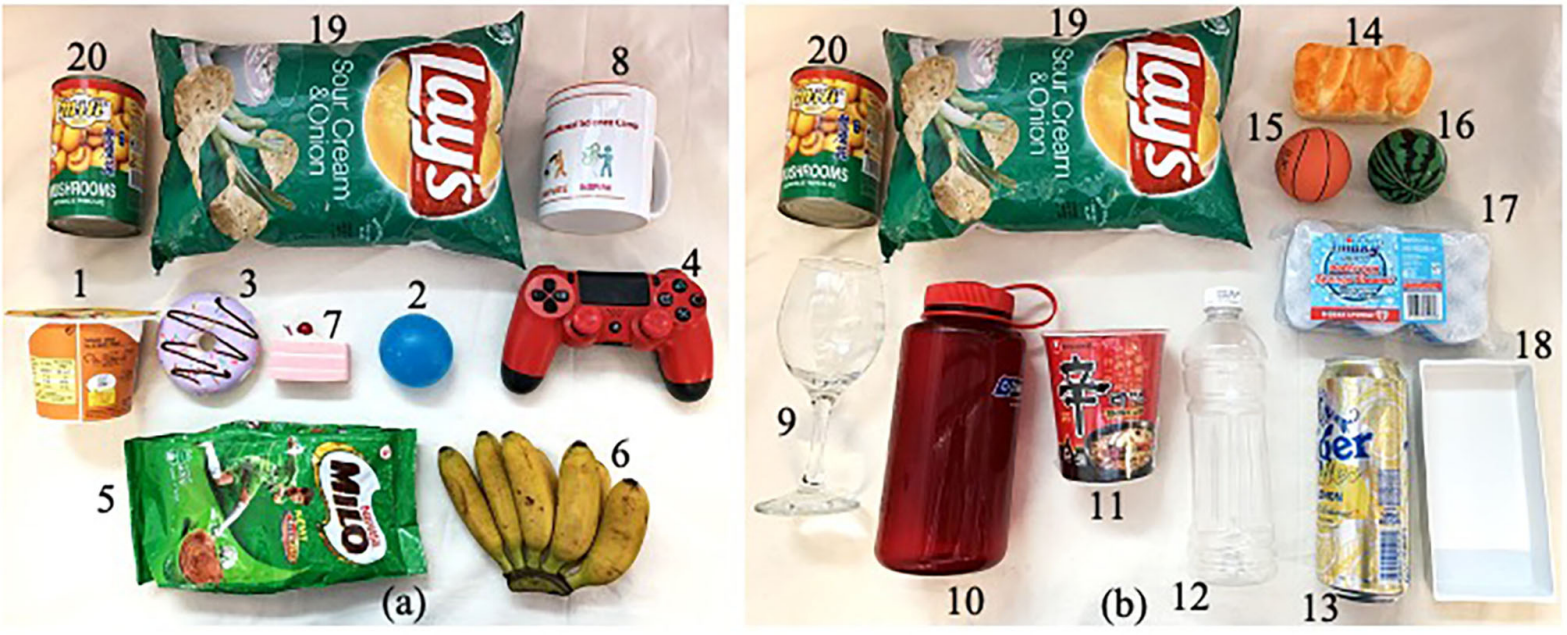
Twenty objects that were used as grasping objects. **(a)** First set of 8 objects with 2 reference objects, **(b)** Second set of 10 objects with 2 reference objects.

To collect the data, soft robotic hand was integrated on a collaborative robotic arm (UR 5e) (Universal Robots, Odense, Denmark). This paper attempts to develop a data-driven grip stability estimation network which learns to recognize stability of object within the grasp of the robotic hand. Force sensing mechanism would be used to distinguish instances whereby the object is merely shifting or tilting within the hand or if the object is slipping from the hand. Twenty different objects were used as grasping items (Figure 5). The choice of items covers a range of features: rigidity, fragility, shape, size and weight. For instance, bag of refill powder and chips are deformable as their shapes change upon being grasped. This makes the grasping force profile more unpredictable as compared to objects with rigid surface such as wineglass. It is challenging to apply the right amount of pressure to manipulate these items with deformable packaging and yet not damage the food items inside. Items such as wineglass are considered as delicate items and are prompted to be damaged by rigid gripper without proper force control. This underscores the usage of soft robotic hands which provide compliant grasping interface and sufficient grasping strength to lift the items without damaging them.

Instead of grasping and lifting the objects using a single grasp pose, each of the object was grasped using one suitable pose from a selection of four poses (**Figure 7**). These four grasp pose were curated because power grasp pose (Pose 1) would not be a suitable choice to grasp all the 20 objects. In particular, objects with smaller profile could be grasped well with fewer fingers (Pose 2 or Pose 3). Objects which are shorter and wider, i.e., banana, could be grasped with power grasp pose. However, it would naturally be more suitable for the soft hand to approach the banana from top-down orientation (Pose 4). Using the selected grasp pose, each object was held on the table for 1.5 s and lifted 50 mm above the table in 1 s. For each object, the experiment was repeated 15 times whereby supplied pneumatic pressure was varied to 50, 80, and 120 kPa for each of the five trials. Hence, there are 15 sets of time series based force sensor readings (15 grasping trials) for each object at the three different supplied air pressures and a total of 300 sets of time series based force sensor readings (300 grasping trials) for 20 objects at three pneumatic pressure.

An Inertial Measurement Unit (IMU) (Pololu, NV, USA) was attached on the object and stability of object was monitored by tracing the acceleration of IMU in three axis. Time series of IMU sensor readings and embedded force sensor readings were continuously recorded at 100 Hz which is within the range of 50–500 Hz of mechanical transients sensing by fast adapting type I (FA-I) and fast adapting type II (FA-II) tactile afferents in human skin sense (Romeo and Zollo, 2020). In post processing, rate of change of IMU acceleration in three axis (Ax, Ay, and Az) were analyzed. The object was considered to have shifted when the rate of change of Ax, Ay, or Az exceeds 5%. During lifting, movement of objects due to grasp instability may occur. If the object began to slip from the hand, subsequent fluctuation in acceleration would be observed. Based on this, force sensor recordings were labeled in three classes based on movements of the object. The three classes are (1) object is stably grasped by the hand, (2) object is grasped by the hand and it is tilting or slightly shifting within the hand, (3) object slips from the hand.

### Development of Grip State Estimation Networks

**Figure 6:**
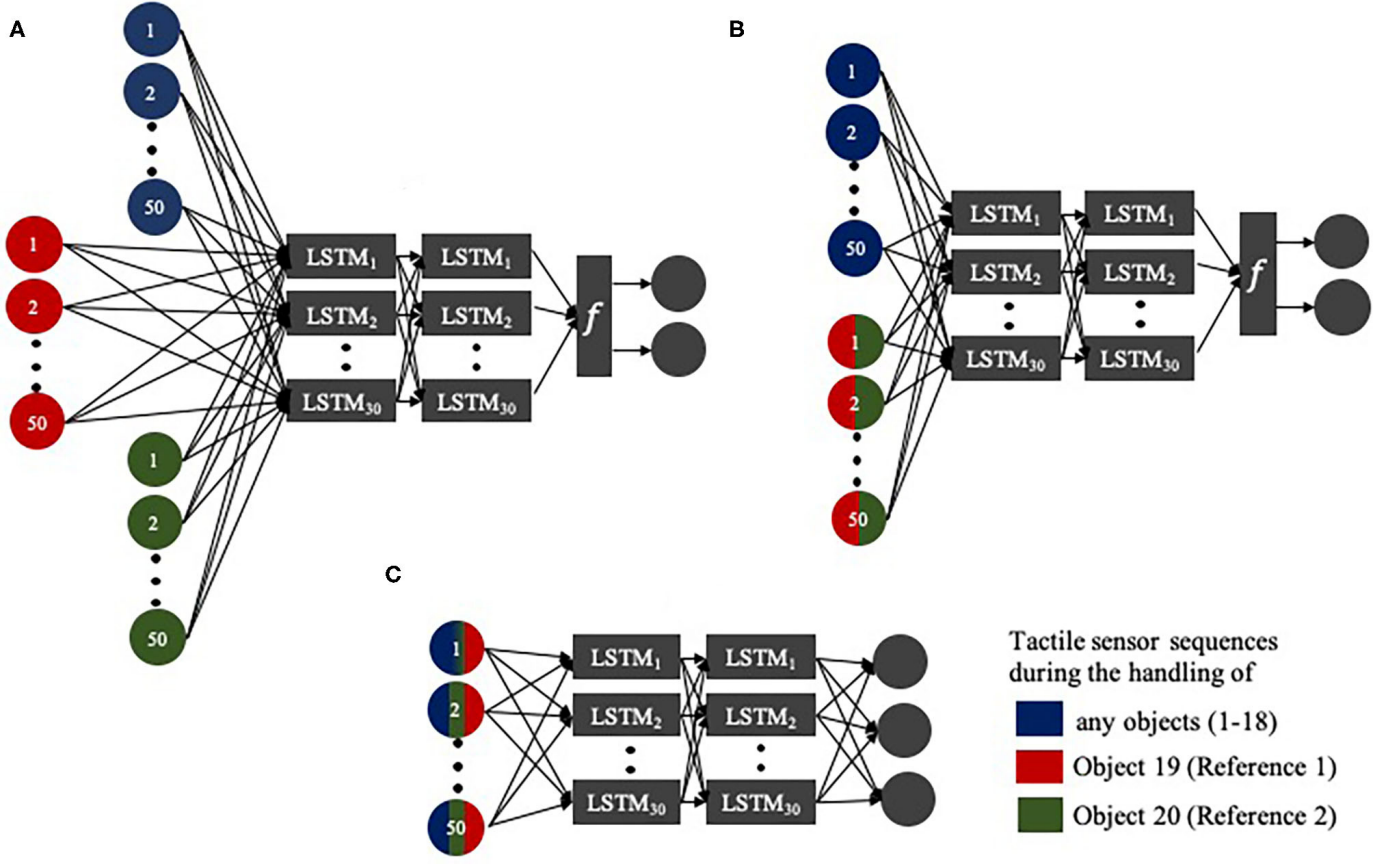
**(A)** Triplet LSTM network, **(B)** Siamese LSTM network, **(C)** LSTM network. Network receives inputs from different objects as indicated by the colors. Multi-colored input bubbles indicate that the network receives sensor readings from either one of the indicated objects.

Three types of networks were developed and their grip state estimation accuracies were compared (Figure 6). The first network is two layered Long Short-Term Memory (LSTM) recurrent neural network which has 30 neurons per layer. The choice of LSTM is due to sequential nature of contact force readings that are obtained from the embedded sensors. Second network is a Siamese LSTM network which also has two layers of neurons with 30 neurons per layer. Siamese network is made of twin networks which simultaneously receive two different inputs and share weight matrices (Bromley et al., 1993). At the top of the network, a function is implemented which receives the high level feature representations of the two distinct inputs for further computation. The third network is a triplet network which also has two layers of neurons and 30 neurons per layer. Triplet network functions similar to the second Siamese network but it has an additional parallel layer of network which allows the network to receive three distinct inputs.

The first LSTM network receives only one input of force sensor readings. It is noted that force readings of different grasping trials were independent of each other. Hence, the network was trained episodically as it was repeatedly provided with different sets of sensor readings from grasping trials. The time series data of different grasping trials were not concatenated. For the first set of 10 objects (objects 1–8, 19, & 20), four sets of force sensor readings (four grasping trials) at each pressure level (total of 12 sets of force sensor readings) were used to train the network with 20% validation split. For this set of objects, grip state estimation accuracy was tested using the remaining grasping trial data. Then, for the next set of 10 objects (object 9–18), one set of force sensor readings (one grasping trial) at each pressure level (total of 3 sets of force sensor readings) were provided as input. The grip state estimation accuracy was tested with unseen force readings from different grasping trials. LSTM network has an output layer with three neurons which predicts the stability of objects based on three classes as mentioned previously. The output layer has softmax activation function and is trained with categorical cross entropy loss function.

In computer vision, sample data were paired to be “similar” or “dissimilar” for Siamese networks. In the case of triplet network, triplet of data would be coupled as “anchor,” “positive,” and “negative” samples (Bromley et al., 1993). However, in this scenario, manipulation process of objects by the soft hand was allowed to occur naturally. Occurrence of slippage or object instability were not manually orchestrated. Thus, labeling of force sensor readings from different grasping trials have become more indistinct as one set of force sensor readings would have occurrence of grasped object instability or slippage at random time interval. However, comparison study for Siamese and triplet networks can still be achieved by selecting grasp objects which have high failure rates and high success rates, to be used as references for comparison.

**Figure 7:**
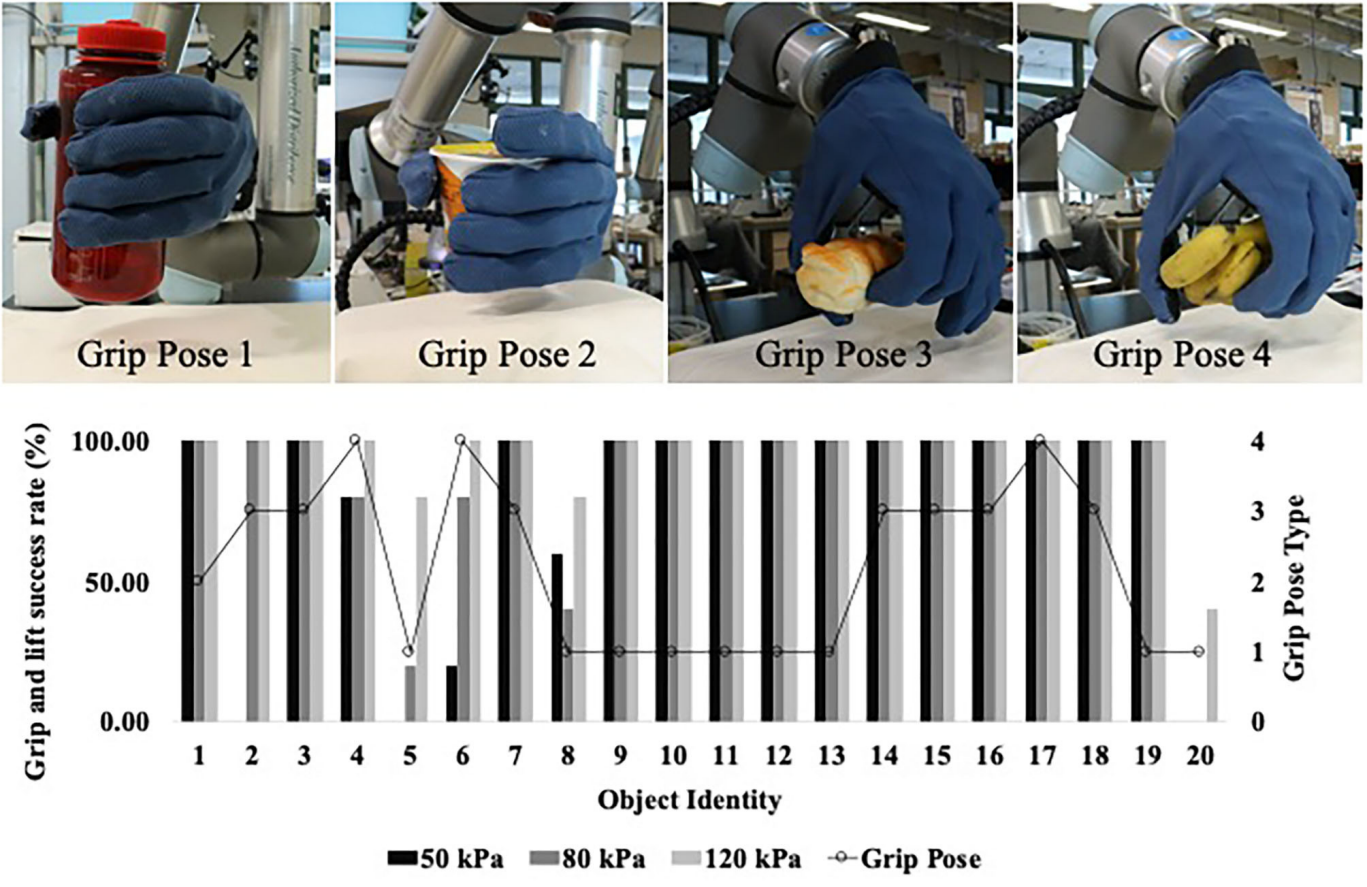
Grasp and lift success rates of 20 objects out of five trials and the type of grasp pose that were used to pick up the object.

To further elaborate, two objects (object 19 and 20) were chosen to be used in comparison learning with the remaining grasp objects. Based on Figure 7, it is noted that object 19 exhibited one of the highest grasp success rate at all pneumatic pressure level and object 20 exhibited lowest grasp success rate at all pneumatic pressure levels. However, as no two sets of samples would be completely similar or dissimilar to each other, these two objects would simply be referred to as “reference” objects. The remaining 18 objects would be referred to as “query” objects. Siamese and triplet networks determine the similarity or dissimilarity between query object and reference object based on smaller batches of time series which have lookback window of 50. Time-series based LSTM networks were reported to improve accuracy with window size up to 50, beyond which the increase in window size does not produce significant improvement (Wyk and Falco, 2018). By learning to compare with objects of high success rate (object 19) and highest failure rate (object 20), the network learns to estimate the stability of query objects.

Siamese network was trained in same manner as described above for the first LSTM network. However, due to pairing of input data, the first set of objects would only include eight items (object 1–8). For instance, Siamese network is first trained with a pair of force sensor readings from object 1 and object 19 (reference 1). In the subsequent cycle, the network receives a pair of sensor readings from object 1 and object 20 (reference 2). Thus, the network always receives two different pairs of sensor readings for the same query object (i.e., object 1) with either one of the two reference objects (object 19 or 20). For the second set of objects, the networks would receive one set of force sensor readings at each pressure level from each query object. These input data would also be compared with that of reference objects (object 19 and 20). The network was tested using unseen sets of force readings from remaining grasping trials for the 18 objects. The Siamese network has an output layer with two neurons which predict whether the query sample is similar or dissimilar to reference sample. The output layer has softmax activation function and is trained with categorical cross entropy loss function.

Similarly, triplet network receives a triplet of data which consist of force sensor readings from selected grasping object, reference 1 (object 19) and reference 2 (object 20). Triplet network was also trained in the same episodical manner as Siamese network. The network was also tested using unseen sets of force readings from remaining grasping trials for the 18 objects. However, the network is able to simultaneously receive input of sensor readings for query object (i.e., object 1) and two reference objects. Triplet network has an output layer with two neurons which predicts whether the query sample is similar or dissimilar to each reference sample. The output layer has sigmoid activation function and is trained with binary cross entropy loss function.

In Siamese and triplet LSTM networks, two different lambda functions were embedded between final LSTM layer and output dense layer. Lambda function implemented in Siamese network is as follows:

(1) $$\begin{array}{l} D_{S}=\;f\left(x_{q}\right)-f\left(x_{ref}\right) \end{array}$$

This function compares the similarity between the two samples in a pair by measuring the distance between encoded features of query samples (*f*(*x*_*q*_)) and reference samples (*f*(*x*_*ref*_)). The lambda function that was implemented in triplet network, was implemented as follows:

(2) $$\begin{array}{l} D_{T}=\left(\;f\left(x_{q}\right)-f\left(x_{ref1}\right)\right)-\left(\;f\left(x_{q}\right)-f\left(x_{ref2}\right)\right) \end{array}$$

The function measures the similarity of query samples (*f*(*x*_*q*_)) to reference sample 1 (*f*(*x*_*ref*1_)) and reference samples 2 (*f*(*x*_*ref*2_)). It then compares the extent of similarity between the two pairs (*f*(*x*_*q*_) and *f*(*x*_*ref*1_), *f*(*x*_*q*_), and *f*(*x*_*ref*2_)).

## Performance Evaluation

### Characterization of Anthropomorphic Soft Robotic Hand

**Figure 8:**
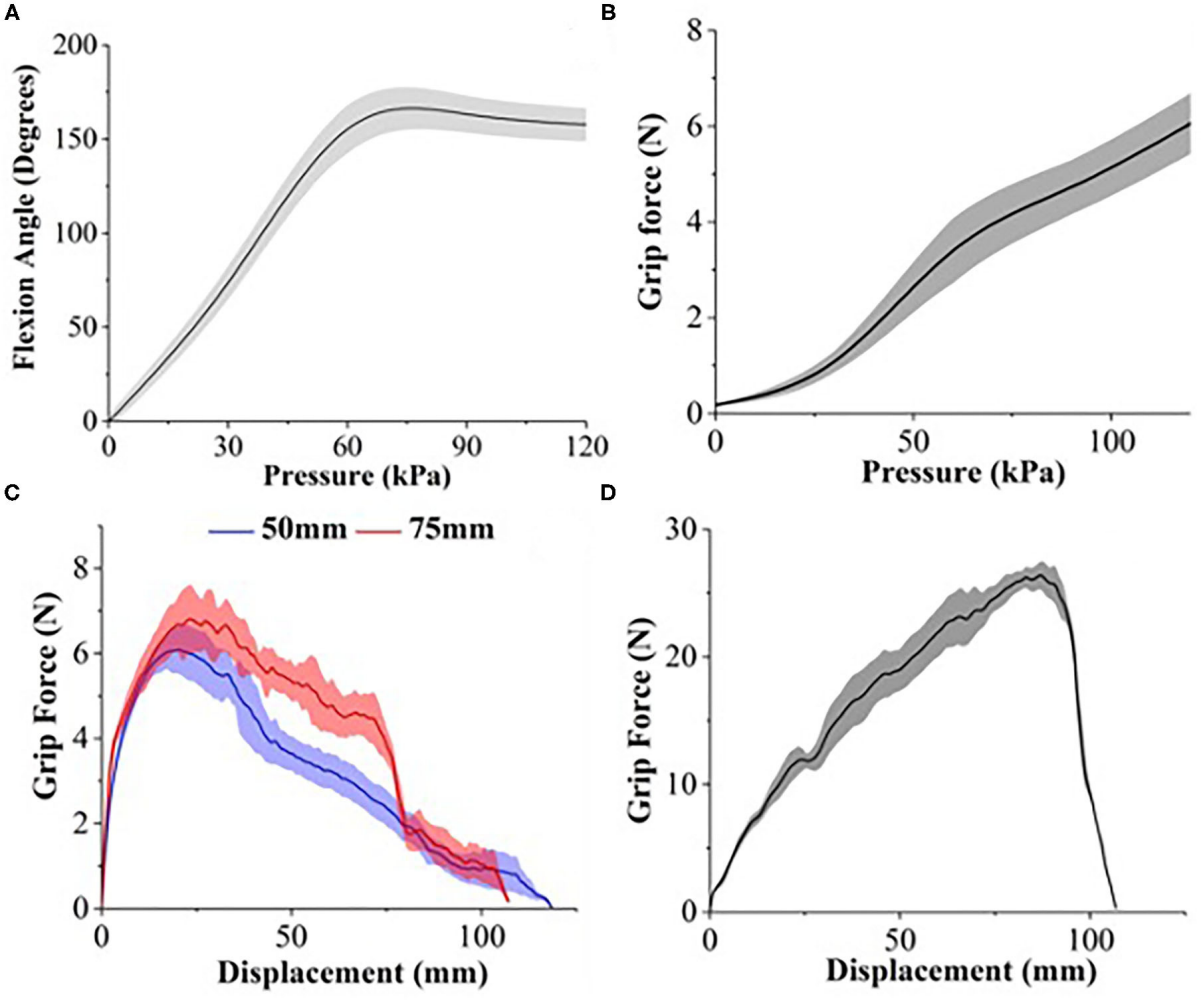
**(A)** Bending profile of FFA. Grip force characterization of **(B)** single FFA in vertical resistive grip test and soft robotic hand in **(C)** horizontal resistive grip test and **(D)** vertical resistive grip test.

During the depressurized state, FFA has a bending angle of 112.33 ± 3.95° (baseline). The actuator achieves maximum bending angle when the supplied pressurized air reaches the range between 60 and 80 kPa (Figure 8A). When pneumatic pressure is further increased, the bending angle of FFA starts to decrease. The maximum bending angle, as shown in Figure 8A, is 166.39° at 75 kPa and reduces to 157.76 ± 8.86° at 120 kPa. The irregularity in the bending profile of the FFA is due to the interaction between the backing layer and the inflating flexion actuator. A considerable straightening effect is observed in flexion of the actuator when the pneumatic pressure increases beyond 75 kPa.

As described, the flexion actuator is folded in pleats and secured in place with a backing layer of fishbone shaped TPU-coated fabric. These placeholders fixate the longer folded air bladder on top of the shorter air bladder. This patterned fixture design reinforces the manifestation of flexion behavior of the FFA when the upper folded air bladder is pressurized. However, when the pressure increases to a certain value (in this case, 75 kPa), the restricted segments of the folded air bladder stiffen and begin to straighten the folded actuator into a beam. When pneumatic pressure increases beyond 75 kPa, equilibrium state is reached between the resistive force exerted by the stiffening folded bladder and the restraining force applied by the placeholders of reinforcement backing layer.

The vertical resistive grip force of FFA increases exponentially against the air pressure (Figure 8B). The maximum grip force is 6.05 ± 0.63 N at the maximum pressure of 120 kPa tested in this study. Compared to the high-force 3D-printed single-channel actuator (Galloway et al., 2016), it requires smaller air pressure to achieve similar grip performance. The 3D-printed actuator requires air pressure of 200 kPa to withstand up to 6 N of vertical pulling force.

The maximum horizontal resistive grip force generated by the soft robotic hand (Figure 8C) at 120 kPa is 6.2 ± 0.38 N and 7.1 ± 0.58 N, with a cylinder of diameter 50 and 75 mm, respectively. The maximum vertical resistive grip force generated by the soft robotic hand (Figure 8D) at 120 kPa is 26.53 ± 0.87 N with a cylinder of diameter 50 mm. These results show that the grip strength of the soft robotic hand is comparable with other soft pneumatic robotic grippers. A gripper with two silicone-based bellows-type actuators generates 16.6 N vertical resistive grip force and 5.6 N horizontal resistive grip force at 124 kPa operating air pressure under the same testing condition (Galloway et al., 2016).

On the other hand, it performs better than soft robotic grippers using other types of actuators such as shape memory alloy-based actuators and dielectric elastomer actuators. For instance, a three-fingered high stiffness shape memory alloy-based gripper can only generate 5.8 N of vertical pulling force when holding a test cylinder of diameter 80 mm (Wang and Ahn, 2017).

### Analysis of Grip State Estimation Networks

Three LSTM based networks have been developed and were trained to estimate the stability of grasped object during the holding and lifting action of the robot. Based on the physical form of the object, it will experience instability in terms of slight shifting or tilting in position when it is lifted from the table. In certain scenarios such as insufficient grip strength, the object will tilt and then slip from the hand as the robot attempts to lift it up. Most of the 20 objects can be gripped and lifted successfully at three pneumatic pressure levels (50, 80, and 120 kPa). However, there are exceptions such as object 5, 6, and 20 which require higher grasping strength to be lifted.

**Figure 9:**
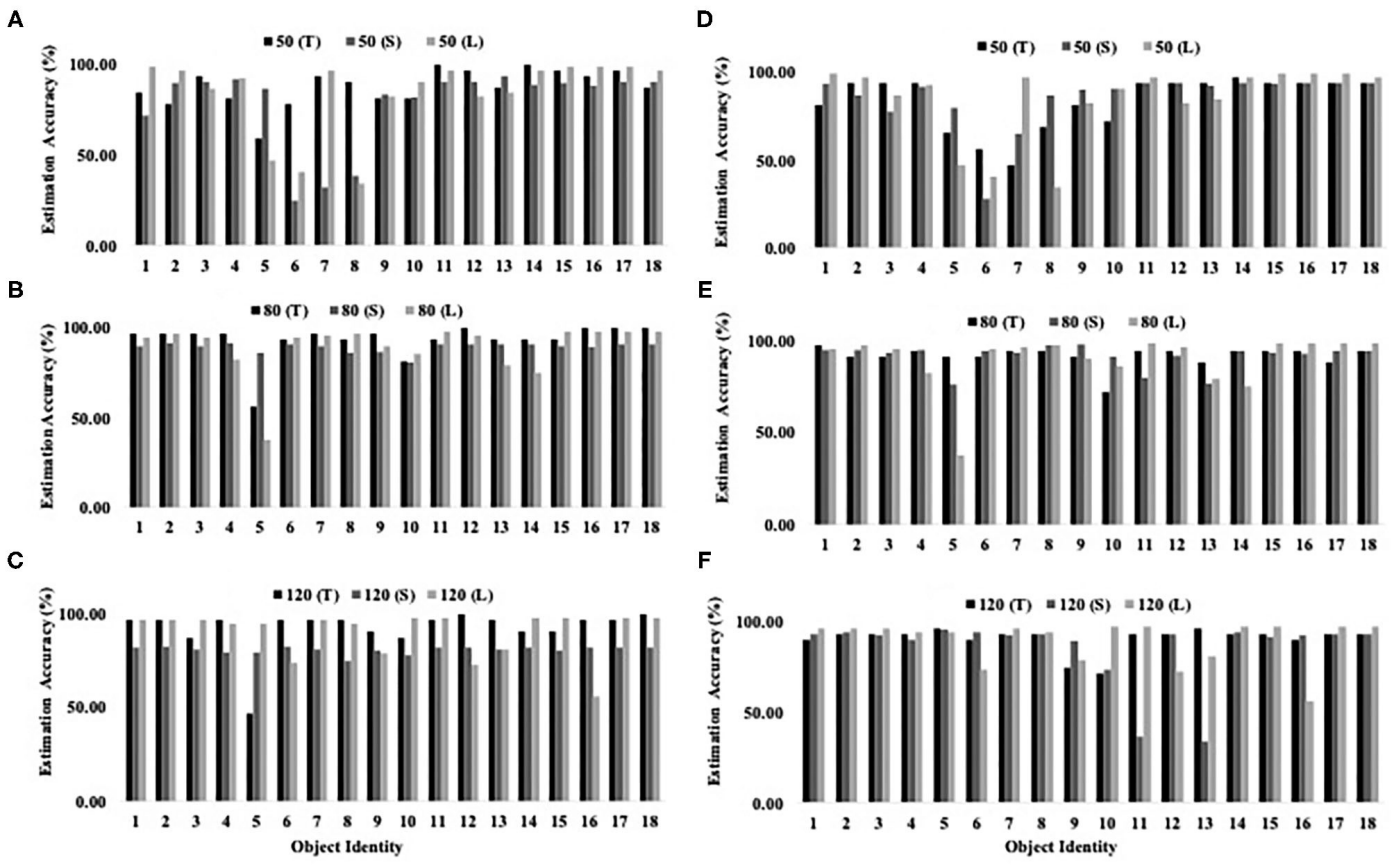
Estimation accuracy of grasped object stability by three networks (T, Triplet network; S, Siamese network; L, LSTM network) at three different pneumatic pressure (50, 80, and 120 kPa). Graph **(A–C)** were produced by comparing the query objects with reference object 19. Graph **(D–F)** were produced by comparing the query objects with reference object 20.

Figure 9 shows the grip state estimation accuracy of three networks on 18 objects at three different pneumatic pressure levels. Object 19 and 20 were excluded as they were used as comparison references in Siamese and triplet networks. The results show that the networks are generally able to accurately estimate the stability of object that has been grasped and lifted by the hand. It is noted that at lower pneumatic pressure (50 kPa), estimation accuracy decreased. This could be attributed by the lower grasping strength of the robotic hand which affected the accuracy of contact force readings by the embedded sensors. It is also noted that it is challenging to estimate the stability of object 5 which is a deformable bag filled with powder. The change in shape of object 5 varies across different trials and hence, this would also increase variation in contact force readings for different trials. Hence, this limits the ability of network to accurately predict the stability of object 5.

Among the three LSTM networks, triplet network achieved overall stability estimation accuracy at 89.96%, followed by single LSTM network with 88.00% and Siamese LSTM network with 85.16%. Compared to single and Siamese LSTM networks, the triplet network received one query sample and two reference samples. This allowed the network to simultaneously compare the query sample with two contrasting references which enabled it to distinguish the stability status of objects more efficiently. On the other hand, Siamese LSTM network did not perform as well-even though it received one query sample and one reference sample. The lambda function in Siamese network measures the distance between encoded features of query sample and reference sample. While easy negative samples can be differentiated easily, hard negative samples may be placed closer to the query samples than positive reference samples (Schroff et al., 2015). As the Siamese network is unable to simultaneously compare the query sample with both references, it is not able to effectively distinguish the status of object stability. The aforementioned grip state estimation accuracy of 89.96% remains comparable with 82.88–91.71% grasp success detection accuracy reported by Zimmer et al. (2019).

## Conclusion

To conclude, we introduced development of fabric based anthropomorphic soft robotic hand. By leveraging on the integrated force sensors, this paper contributed by developing one shot learning based grip state estimation networks which interpret the stability of objects that have been grasped and lifted using the soft hand. The developed networks learnt to estimate the following occurrence during the manipulation process- (1) object is stably grasped by the hand, (2) object is grasped by the hand and it is tilting or slightly shifting within the hand, (3) object slips from the hand. During the lifting, object may tilt or shift slightly due to acceleration of the robot or due to grasping strength of the soft hand. Heavy objects which were grasped with insufficient grasping strength, may subsequently experience slippage. We have curated a diverse range of 20 grasping objects in terms of rigidity, fragility, shape, size, and weight. For instance, a plastic bag of refill powder and chips are deformable as their shapes changes upon being grasped. This makes the grasping force profile more unpredictable as compared to objects with rigid surface such as wineglass. The developed networks were primarily trained with 10 objects whereby four sets of force sensor readings at each pressure level were provided as input. For the next set of 10 objects, the number of required samples were reduced to one set of force readings at each pressure level per object. Nonetheless, the networks are still able to estimate stability of grasped objects at an average of 85.16–89.96%. Hence, this study has showed that one shot learning which has mostly been used in machine vision, could be adopted to develop tactile perception in sensorized soft hand. In future study, the networks could be further improved and integrated with pneumatic control system as real time feedback system.

## Data Availability Statement

The original contributions generated for the study are included in the article/supplementary material, further inquiries can be directed to the corresponding author/s.

## Author Contributions

All authors contributed equally to the manuscript preparation, data analysis, and experiments presented in the paper.

## Conflict of Interest

The authors declare that the research was conducted in the absence of any commercial or financial relationships that could be construed as a potential conflict of interest.
